# Perceptions and Attitudes towards Medical Research in the United Arab Emirates: Results from the Abu Dhabi Cohort Study (ADCS) Focus Group Discussions

**DOI:** 10.1371/journal.pone.0149609

**Published:** 2016-03-04

**Authors:** Yusra El Obaid, Aisha Al Hamiz, Abdishakur Abdulle, Richard B. Hayes, Scott Sherman, Raghib Ali

**Affiliations:** 1 Institute of Public Health, United Arab Emirates University, Al Ain, United Arab Emirates; 2 Public Health Research Center, New York University Abu Dhabi, Abu Dhabi, United Arab Emirates; 3 Department of Population Health, New York University School of Medicine, New York, New York, United States of America; The George Washington University School of Medicine and Health Sciences, UNITED STATES

## Abstract

**Background:**

In developing medical research, particularly in regions where medical research is largely unfamiliar, it is important to understand public perceptions and attitudes towards medical research. In preparation for starting the first cohort study in the United Arab Emirates, the Abu Dhabi Cohort Study (ADCS), we sought to understand how we could improve the quality of the research process for participants and increase public trust and awareness of research.

**Methods:**

We conducted six focus groups (FG), consisting of Emirati men and women aged above 18 years to resemble the target population for the ADCS. Sampling was purposive and convenient. Data collection was an iterative process until saturation was reached with no new themes identified. Text from each FG was analyzed separately by identifying emerging issues and organizing related concepts into categories or themes. A coding tree was developed, consisting of the main concepts, themes, subthemes and corresponding quotes. Both themes and main ideas were identified using inductive analysis.

**Results:**

Forty-two participants enrolled at 3 academic centers (New York University Abu Dhabi, UAE University, Zayed University) and the Abu Dhabi blood bank. Focus group participants described lack of awareness of research as a challenge to participation in clinical research studies. Altruism, personal relevance of the research, and the use of role models were commonly identified motivators. Participants were generally satisfied with the informed consent process for the ADCS, but would be disappointed if not provided test results or study outcomes. Fear of a breach in confidentiality was a frequently expressed concern.

**Conclusions:**

Participants join research studies for varied, complex reasons, notably altruism and personal relevance. Based on these insights, we propose specific actions to enhance participant recruitment, retention and satisfaction in the ADCS. We identified opportunities to improve the research experience through improved study materials and communication to participants and the broader community.

## Introduction

The World Health Organization’s declared in 2004 that: *‘Well planned health research is fundamental to the improvement of health in all countries*.*’* [1] However, countries in the Arab world (particularly those in the Gulf Co-operation Council), despite having made rapid economic progress, have generally struggled to conduct sufficient high-quality research due to a lack of resource allocation, human capacity, and limited public participation in clinical research. [2]

The United Arab Emirates (UAE) has one of the best healthcare systems in the region and has made significant progress in establishing major academic and research institutions, yet the region is now facing major public health challenges with increasing life expectancy and changes in lifestyle leading to a large and rapidly increasing burden of chronic diseases, particularly obesity, diabetes and cardiovascular disease. [2] Research is needed to address these challenges, however; there have been no prospective cohort studies to date in the UAE and so the relative importance of both established and novel risk factors for these common chronic diseases in the region is largely unexamined. To address this research gap, we are developing a prospective cohort study of UAE nationals, *the Abu Dhabi Cohort Study*, to determine how the health of Emiratis is affected by their lifestyle, environment and genes, with specific attention to risk factors for obesity, diabetes and cardiovascular disease.

In developing research in the UAE there is a significant need to consider cultural context, however; little is known about the factors, cultural or otherwise, that can promote public participation in medical research in this population. Such information may be valuable to improve our ability to conduct major cohort studies through increasing rates of recruitment and retention [3]. Of particular importance is the general public perception, willingness and attitudes to participate in medical research [4]. Investigating attitudinal factors affecting public willingness to participate will ultimately facilitate broad-based recruitment to medical research studies and, in turn, increase the quality and generalizability of study findings.

Because no firsthand information exists as to how participants in the UAE perceive medical research and what motivates them to participate, particularly in longitudinal studies where follow up is a key feature, we conducted focus group (FG) discussions among Emirati men and women as an initial step to understand potential participants’ views of the study and to optimize the participant materials to be used in the Abu Dhabi Cohort Study. The study objectives were to 1) Enhance human subjects’ protection in the Abu Dhabi Cohort Study, including informed consent; 2) Enhance recruitment and retention in the study; 3) Improve the quality of the research process; and 4) Increase public trust and awareness in the research enterprise.

### Aims and objectives

The study’s specific aims were to conduct 6–10 FG s with research participants who resemble the target population of the Abu Dhabi Cohort Study (ADCS) to explore their general perceptions towards medical research and to optimize the participant materials to be used in the study including consent form procedures, invitation letters, participant information leaflets and description of the study process. Prior to conducting the actual discussion, participants were asked about their language preference. Given that all of them were fluent in English, all FG discussions were conducted in English.

## Subjects and Methods

The ADCS participant materials i.e., patient information leaflet, consent form, and other communication material were carefully reviewed by a committee made up of two principal investigators and two research assistants. A set of separate questions in line with the project aims and objectives were prepared for the FG discussions. The committee refined the study design with the benefit of expert input from professionals in human subject research from New York University in New York (NYU), and in Abu Dhabi (NYU-AD). Letters of invitation describing the study objectives and design were sent by email or smartphone to a selected number of students and staff from four institutions (NYU-AD, UAE University, Zayed University (ZU) & The Abu Dhabi Blood Bank (ADBB). Those four institutions were chosen as this is where participants were going to be recruited from for the cohort study, and potential participants were invited on the basis that they were eligible to take part in the cohort study (i.e. that they were UAE nationals and aged above 18).

### Recruitment

Ten to twelve eligible research participants were invited to attend each FG. Each FG was conducted with a minimum of 5 participants. Each FG was conducted in the presence of at least two researchers acting as a group moderator and an observer.

### Ethical approval

This study was approved by the research ethics committees of New York University Abu Dhabi, Abu Dhabi Blood Bank, Zayed University and United Arab Emirates University and participants provided written informed consent.

### FG Discussions

FG s lasted 90 minutes and followed a semi-structured interview process according to the formats described in the discussion guide (Table 1). The moderator validated the main themes of the FG discussions, by restating them at the end of the session and requesting participants to confirm or modify them. FGs were audiotaped and the observer took written notes. Two researchers from the committee held a debriefing session after each FG, during which they identified and discussed the main themes of the FG.

**Table 1:** Discussion topics for FGs.

1. Reasons participants join research studies
2. General understanding of the research design (cohort) and the need for follow-up.
3. Preferred means of communication for follow-up
4. Understanding of genetic research
5. Evaluate and assess the usability of Participant information leaflet and consent form (content, order of discussion, headings, general understanding)
6. Opinions on the proposed recruitment approaches
7. Opinions on the study visit itself (questionnaires, physical assessment, biological tests, samples)
8. Expected positive and negative aspects of the research experience
9. Reasons participants remain in research (specific & general)
10. Reasons participants drop out of research (specific & general)
11. Family involvement and familial reaction to participation
12. Expectations of participants and feedback of results
13. Suggestions for making research participation easier
14. The role of incentives

### Audiotapes and Transcripts

Moderators used open-ended questions to ensure participants fully had a chance to air their views. We stopped conducting focus groups when we reached saturation and no new themes were emerging. Audio-recorders were then switched off and kept securely with the senior moderator. Names, institutions, and other identifiers were redacted from all transcripts, observer notes, and debriefing notes before analysis. FG transcripts were analyzed systematically by coding the data into emerging themes. Coding involved careful reading of all text and documenting passages that related to the main ideas and themes that emerged. The discussion guide was used as a framework for data analysis. Participants’ discussion points were grouped according to topics defined in the discussion guide. Unique perspectives on key issues were also included in the findings. Direct quotes from participants were referenced where useful, to illustrate and support the findings. Themes were identified to represent ideas that emerged. Members of the Committee, as content experts, reviewed examples of the thematic analysis to align and validate the coding strategy. Text from each FG was analyzed separately, first by identifying individual issues that emerged, then organizing related concepts into categories or themes. A final index system was developed, consisting of the main concepts, themes, subthemes and corresponding quotes. Both themes and main ideas were identified using inductive analysis. The purposes for using an inductive approach are: (a) to form a brief, summary format from a rich textual data; (b) to establish clear links between the research objectives and the summary findings derived from the raw data; and (c) to develop a framework of the underlying structure of experiences or processes that are evident in the raw data. The general inductive approach provides an easily usable and systematic set of procedures for analyzing qualitative data that can produce reliable and valid findings.

The transcripts and analyses are provided as S1 File.

#### Results

Six FG s were conducted from September to November 2014 across the four institutions. In total, 42 subjects participated in the 6 FG s– 3 groups were only females, 1 group was only males, and 2 were mixed. Sub-themes identified are illustrated in Table 2.

**Table 2:** Sub-themes identified relevant to invitation, informed consent and participant information leaflet.

1. Extent of satisfaction with the process
2. Time spent reading information
3. Adequacy and clarity of information
4. Individual approach to obtaining consent (style)
5. Extent to which characterization of risks is clear
6. Length of the process and repetition of content
7. Indifference to content of informed consent document
8. Instilling fear of study participation
9. Undue pressure to enroll
10. Adaptation of information to the individual
11. Use of video media

### Themes

#### “Research” is an unfamiliar concept

Most participants (80%) expressed unfamiliarity with the term “medical research”. None of the FG participants had participated previously in any medical research. However, most subjects (80%) also reported that they trusted the medical institutions in the country and did not express any concerns about research participation, as such.

#### Community awareness

Participants suggested many solutions which could increase community awareness of the study objectives and expected positive outcomes. Among the suggested solutions was to advertise through local media, such as TV, radio and newspapers. A particularly important suggestion was to mention the research at mosques at Friday prayers, which are attended by the majority of men in every community. It was also suggested that using current clinical facilities in the community could be an appropriate venue for community outreach programs. A popular approach identified by participants was the use of social media (i.e. Twitter, Facebook, Instagram), as these means of communication are very popular, particularly among young adults. Other participants supported the use of public figures or role models in the community (i.e. sheikhs (leaders), actors, comedians, and heads of health departments in the country) to convey the aims and benefits of the study, as they believed it would add to the credibility of the study and draw the attention of the community. Participants in the study believed that their friends and family would perceive their participation as positive and, therefore, might be encouraged to participate as well.

#### Motivations for participation in research

Participants identified reasons for participating in medical research, as shown in Table 3. Participants most often identified multiple reasons for participation. Altruism was commonly stated as a reason to participate, along with relevance to the individual’s health or the health of family and friends, as well as the anticipated benefits to the community. Incentives and financial motivation were not identified as reasons to participate. Participants identified other forms of incentives like invitations to public events or social gatherings as a good way to attract participation. Some participants admired the uniqueness of the study to the UAE community. Patriotism to the country and national obligation toward the community were reasons for participation. In addition, some participants had the perception that enrolling in research will give them the opportunity to have a free medical checkup and to reassure them of their good health.

**Table 3:** Reasons Identified by Participants for Participating in Clinical Research.

1. Altruism
2. Study topic relevant to the individual’s health or the health of family/friends
3. Anticipated benefits to the community
4. Patriotism to the country and the national obligation toward the community
5. Anticipated learning about science, research or health topics from participation
6. Commitment to volunteerism
7. Free healthcare/check up

#### Challenges to participation

A small proportion of participants (10%), especially females, expressed concern regarding possible confidentiality breaches resulting from participation and mishandling of confidential medical information. Others (20%) pointed out that the ADCS provided limited benefit to the individual, as individual test results were not intended to be shared with participants. An important challenge conveyed by most of the participants, especially females, was finding the time to participate in the study. Some females also mentioned social and cultural barriers to participation, especially if they have to visit the medical study center after work hours.

#### Fear of the unknown

Some participants (24%) expressed their fear of discovering that they have a disease if they participated in the study, as they felt it was better not to know. They also expressed worries that they have to change their life style if they were found to have a disease. Other participants felt that they will be followed up until they develop the disease; one participant stated “when I participate in this study, I will get the disease”. This belief might discourage them from participation.

#### Invitation, participant information leaflet & Informed Consent

The initial invitation email was felt to be too long and not attractive enough to encourage them to open and read the email. Participants also commented on the order and arrangement of the headings and sub-headings of the invitation email. They suggested reducing the amount of text and indicated that the use of logos and pictures would be more attractive. Some participants (10%) also felt that the participant information leaflet (PIL), which includes information required for informed consent, was too long and complex. Most participants (80%) raised the point that the PIL used complicated Arabic language and had repeated explanations, and they suggested it be shortened and simplified. Many participants (50%) suggested the use of visual materials (i.e. videos) to convey the same message in a more accessible and attractive way. Some participants (15%) complained about not fully understanding how much of their time would be required to participate in the protocol. Some participants (15%) felt that the detailed risk explanation and information in the leaflet generated fear and discouraged participation.

In general, research participants were satisfied with the informed consent process. Some participants (10%) stated that they were particularly concerned about study risks and wanted detailed explanations during the consent process. However, others (40%) stated that they trusted the institution to protect their safety. Many participants (50%) appreciated having sufficient time to read the informed consent form and having detailed explanation of study objectives, process and risks and the opportunity to ask questions. Although participants also expressed a desire to have risks clearly defined, this issue was not ranked near the top of participants’ concerns.

#### Human subjects’ privacy and confidentiality

Participants generally trusted the health system in the UAE and had confidence in how their confidential information is handled. However, many participants (50%) expressed concerns about the fear of their confidentiality and privacy being breached if they participated in the cohort study. In general, they didn’t want the research team to access their hospital medical record but said that they might give permission to access their records if their primary physician was part of the research team.

#### Bio-banking

The vast majority of participants (95%) did not mind having their biological samples stored for study purposes and did not have any concerns regarding re-using their bio-medical samples later in future research studies. They didn’t anticipate a need for obtaining a new consent for re-using their samples and they didn’t raise any ethical concerns.

#### Research design and process

Nearly all participants (95%) were unfamiliar with the study design and needed additional explanation regarding the need for follow-up. Some participants identified being followed over time as time- consuming and an additional commitment needed from them. Other participants (10%) described the research team as very optimistic when expecting low rates of dropouts and good retention of study participants throughout the follow-up period. However, some participants (35%) suggested some techniques to minimize drop-out and improve participant retention including: collaborating with health centers across the emirate of Abu Dhabi; encouraging participants to do the follow-up with their primary healthcare provider; limiting repeated investigations and time spent in each visit; sharing some results with participants to increase the sense of ownership and achievement; and, keeping participants regularly updated through organizing social events to enhance their sense of belonging and as a form of appreciation from the research team.

#### Participants’ expectations

Factors associated with participants’ positive expectations are listed in Table 4. Overwhelmingly, the factor most frequently identified as contributing to a positive experience was benefit to the community and the nation (90%). Many participants also expected acquiring new information and learning about health and disease, and some stated that this new knowledge will improve their ability to care for themselves and others in the community.

**Table 4:** Factors Contributing to Participants’ expectations of the Research Experience positively or negatively.

Factors Associated with Positive Expectations	Factors Associated with Negative Expectations
Study involve learning and is interesting	Pain, extended discomfort
Free health monitoring/checkup	Not receiving clinical test results during the study
Feeling valued	Risks, side effects (fears or actual)
Community health improved	Cancellations, waiting
Conquering fear, aversion	Unanticipated aspects of the study (i.e., ‘surprises’)
	Protocol too demanding
	Lack of privacy/ fear of confidentiality breach
	No access to study results
	Undue pressure to stay in the study

The factor most frequently identified (90%) as contributing to negative participant experience and expectations was pain or discomfort, often associated with procedures, such as phlebotomy (especially when these procedures did not proceed smoothly). Cumulatively, issues related to study logistics, such as appointment or procedure delays perceived by the participant as due to poor organization or planning, were also frequently cited as having a negative impact. (Table 4).

The reasons participants identified for remaining in research studies requiring follow up included: staff responsiveness to requests, commitment to the research project, and investment by staff in individual participants. The most common reasons participants thought people would leave research studies were: 1) studies were more demanding than expected; 2) unpleasant side effects, often associated with interventions; and 3) participant inconvenience (e.g., limited clinic hours, difficulty parking, and requirement for numerous visits).

## Discussion

The present study was conducted to assess the general public perception of medical research in the UAE, through focus group discussions among Emirati men and women who represent potential participants in the first cohort study to be conducted in the UAE. Our focus group results showed that although research is a relatively new concept in the community, participants had a positive view of medical research. Potential participation was influenced by different reasons and increasing the awareness of research was thought to positively promote wider community involvement and promotion. Similar to other populations, increasing the awareness of key aspects of the study and improving the overall knowledge of research increases the willingness to participate. [5,6] Participants tended to participate in research studies if they addressed the health concerns of the society. Similar to other studies, it was commonly stated by participants that having a relative or a friend with the disease would encourage them to participate in studies investigating that disease. [7,8] This study suggests that opportunities exist to increase participation by targeting recruitment efforts toward the whole community.

Participants’ satisfaction with the research process was an important factor to encourage participation, as others have reported [9,10]. Conveying a sense of caring for the health of a community and for each person comprising it is thus an integral part of the factors that can positively influence participant satisfaction [11]. Building a research culture in the UAE will help improve follow-up in longitudinal studies and decrease the withdrawal rate, especially given that research is a relatively new concept in the UAE.

Trust between the community and researchers is crucial to the success and continuity of research and in a tribal community like the UAE, using heads of tribes, role models and famous persons to promote research could improve the follow-up in longitudinal studies. Community based participatory research is an evolving concept that involves collaborative work between researchers and community members. It has the ability to generate trust and a positive research culture in the community [7,8]. Allowing community members to share their opinions and contribute to decision making increases their sense of ownership and trust [7,8,11]. Researchers need to understand the social and cultural aspects of their community, which in turn helps to promote participation.

Although the ownership of donated bio-samples is a controversial topic, it is well established by ethics committees that study subjects have the right to decide their present and future use [12]. Our study participants were generally happy to donate biological samples and were not concerned regarding their future re-use, whereas a study in Jordan showed that one third were not willing to have their samples bio-banked [13].

Improving and promoting medical research in the community requires concerted effort from relevant stakeholders, with strategic goals agreed upon by the community decision makers and lead scientists. The strategy should include upgrading the research infrastructure, providing sufficient funds and creating a good reputation for research through training researchers in order to attract and retain active participation in the community. Improving public health requires good evidence, which in turn requires good research.

The challenges for quantitative and qualitative research include the varied composition and mobility of the UAE population, with limited health records and disease registries. Long-term follow-up of participants may be a major obstacle for longitudinal studies. [14] There can also be a reluctance shown by parts of the population to participate in studies, especially those that require responding to what are perceived as sensitive questions. Successful execution of population research in the UAE requires an understanding of socio-cultural aspects of the study population, and good communication between researchers and participants.

### Conclusion

The results of this study support the promotion of health research in the UAE and confirm that establishing a population-based cohort study will make a very positive contribution to building a research culture in the country. It suggests that the community may be best targeted by strategies that enhance communication of the personal and societal health benefits of biomedical research. This study represents an important start to understanding the public experience with research recruitment and retention. Participants join studies for varied, complex reasons, including altruism and personal relevance. Based on these insights, we proposed specific actions to enhance participant recruitment, retention and satisfaction in the Abu Dhabi cohort study; these include using simpler language in the invitation letter and participant information leaflet; ensuring clarity in communication of why research is being done; making effective use of social structures and different media and sharing the results of research with participants.

## Supporting Information

S1 File Focus group discussions—transcripts and analysis. (DOCX)

## References

[1] WHO [Internet]. World report on knowledge for better health: Research policy, meetings and publications; 2004 [cited 2015 October 2]. Available from: http://www.who.int/rpc/meetings/wr2004/en/index14.html.

[2] El-Azami-El-Idrissi M, Lakhdar-Idrissi M, Ouldim K, Bono W, Amarti-Riffi A, Hida M, et al Improving medical research in the Arab world. Lancet. 2013; 382 (9910): 2066–2067. 10.1016/S0140-6736(13)62692-6 24360385

[3] Kost RG, Lee LM, Yessis J, Coller BS, Henderson DK, the Research Participant Perception Survey Focus Group Subcommittee. Assessing Research Participants’ Perceptions of their Clinical Research Experiences. Clinical and Translational Science. 2011; 4(6): 403–413. 10.1111/j.1752-8062.2011.00349.x 22212221 PMC3640854

[4] Trauth JM, Musa D, Siminoff L, Jewell IK, Ricci E. Public Attitudes Regarding Willingness to Participate in Medical Research Studies. J Health Soc Policy. 2000; 12(2): 23–43. 11184441 10.1300/J045v12n02_02

[5] Elger BS, Caplan AL. Consent and anonymization in research involving biobanks: Differing terms and norms present serious barriers to an international framework. EMBO Rep. 2006; 7(7): 661–666. Available from: doi: 10.1038%2Fsj.embor.7400740 16819458 10.1038/sj.embor.7400740 PMC1500833

[6] Lichtenberg P A, Brown DR, Jackson JS, Washington O. Normative health research experiences among African American elders. J Aging Health. 2004; 16 Suppl 5: 78S–92S. 15448288 10.1177/0898264304268150

[7] Christopher S, Watts V, McCormick AKHG, Young S. Building and maintaining trust in a community-based participatory research partnership. Am J Public Health. 2008; 98(8): 1398–1406. 10.2105/AJPH.2007.125757 18556605 PMC2446462

[8] Minkler M, Blackwell AG, Thompson M, Tamir H. Community-Based Participatory Research : Implications for Public Health Funding. Am J Public Health. 2003; 93(8): 1210–1213. 12893597 10.2105/ajph.93.8.1210 PMC1447939

[9] Manafa OU, Lindegger G, Ijsselmuiden C. Informed Consent in a Clinical Trial: Participants Satisfaction of the Consent Process and Voluntariness of Participation. Online Journal of Health Ethics. 2006 3(1). Available from: 10.18785/ojhe.0301.06

[10] Kelley K, Clark B, Brown V, Sitzia J. Good practice in the conduct and reporting of survey research. Int. J. of Quality in Health Care, Oxford University Press. 2003; 15(3): 261–266. 10.1093/intqhc/mzg031 12803354

[11] Israel B A, Schulz A J, Parker E A, Becker A B. Review of community-based research: assessing partnership approaches to improve public health. Annu Rev Public Health. 1998; 19: 173–202. 9611617 10.1146/annurev.publhealth.19.1.173

[12] Ma Y, Dai H, Wang L, Zhu L, Zou H, Kong X. Consent for use of clinical leftover biosample: a survey among Chinese patients and the general public. PLoS One. 2012; 7(4): e36050 10.1371/journal.pone.0036050 22558323 PMC3338618

[13] Ahram M, Othman A, Shahrouri M. Public support and consent preference for biomedical research and biobanking in Jordan. Eur J Hum Genet. 2013; 21(5): 567–570. Available from: doi: 10.1038%2Fejhg.2012.213 22968133 10.1038/ejhg.2012.213 PMC3641386

[14] Aw TC, Zoubeidi T, Al-Maskari F, Blair I (2011) Challenges and strategies for quantitative and qualitative field research in the United Arab Emirates. Asian Pac J Cancer Prev 12: 1641–1645. Available: http://www.ncbi.nlm.nih.gov/pubmed/22126513. 22126513

