# Supplementary material for: Perceptions and Attitudes towards Medical Research in the United Arab Emirates: Results from the Abu Dhabi Cohort Study (ADCS) Focus Group Discussions
Source: PLoS One. 2016 Mar 4;11(3):e0149609. doi: 10.1371/journal.pone.0149609 (PMC4778844; doi:10.1371/journal.pone.0149609)
Supplement: S1 File — (DOCX) [file pone.0149609.s001.docx]

**Analysis (transcript 1, 3 & 7)**

| **Themes** | **Categories** | **Codes** |
| --- | --- | --- |
| Perception of medical research | - Reasons for participation in medical research - Reasons for refusal of participation in medical research | - Altruism - Benefit to the whole country - Study topic relevant to the individual’s health or the health of family/friends - Anticipated learning about science, research or health topics from participation - Importance of the study - discovery of new therapies - fears of confidentiality breach - sensitive information (smoking) and privacy - shyness - time constraints - life commitments - lack of community awareness |
| Fear of unknown | - change - study perception | - fear from unknown, better not to know - People do not want to change their lifestyle for a certain disease. They fear of changes. - people are followed up until they get the disease - That I participate in this study, therefore I will get a disease. And this might put people off. People will be pessimistic about this |
| Publicity | - Increase community awareness | - Take advantage of events happening during the year (national day, thalassemia day) - Use family medicine clinics - TV and newspapers - Brushers and leaflets - Collaboration with junk food restaurants - New generation/ social media - Add the link to the picture in the social media - Use short video (15sec) in Instagram - Use students advisors - Assign study ambassadors in each location - Get public media: social media, youtube, Abu Dhabi TV/radio, Emirates News - Support group to share experiences, if someone in the family had the disease. |
| Research design (cohort) | - Follow up & drop outs | - Time constraint - Pessimistic - Collaborate with health centers to limit drop outs. - Proper study awareness (benefits) urge participants to continue the study - Use phone - Disturbance   travelling/moving  changed their mind- better not to know   - avoid blood tests and other investigations in follow up |
| Confidentiality | - Breach of confidentiality | - Decline permission to access medical records. - Personal stuff - Majority will refuse - Conservative society - Part of culture - Use physicians they trust to supervise your access |
| Genetic research |  | - Well known - Better not to know about the existence of a problem |
| Names and slogans |  | - Name: Hayatna, Our generations, Our lives, my step - Slogan: for healthy future/ our country our future/ our generations our lives, خطوة للصحة, my step for a healthy future - Participants have no children/ doesn’t make sense to put generations |
| Participants Information Leaflet  Email invitation | - Time spent reading information - Adequacy and clarity of information - Level and clarity of language - Extent to which characterization of risks is clear - Instilling fear of study participation - Length of the process and repetition of content - Undue pressure to enroll - Adaptation of information to the individual - Use of video media | - Generates fear - Make people refuse - Strong wording - Complicated - Too much information - Too long - No one will read it - Repetition - Video will stuck in people’s minds - Include how the study would increase the life expectancy of people in the UAE to improve their health. - Explain that in UAE we don’t have this kind of study and it’s the first in the UAE that would encourage people to participate. - They will feel like they are a part of the study - It is not necessary to include the bruising and the natural blood test experience. Everyone knows that - summarize it - repetitive: privacy and confidentiality is the same - last question and the first question are important   long title  Calling them is good   - Having a poster would be good.   listing the benefits of the study in the email |
| Study process | - Lengthy process - Development of chronic disease notification | - Lengthy process - Not answering personal Qs in the questionnaire - Simplify - Too much information required - Require going somewhere for participation - Will generate dis-honest answering - Undue pressure to drop out - study is boring, 40-50% only will continue - Participants will not inform: Last thing to do is to remember the study after developing chronic disease. - Better to get permission from Thiqa or Seha |
| Expectations | - Factors associated with expected positive experiences - Factors associated with expected negative experiences | - Benefits to the country - Study involve learning and enquiring new knowledge - Feeling valued - Community and future generation health improved - UAE will have a strong database - Reading a study that was done in UAE population not US or UK population. - Extended waiting time - Lost to follow up - Lengthy process - Not receiving test results back - Risks, side effects - Unanticipated aspects of the study/ fear of unknown - Protocol too demanding - Breach of confidentiality |
| Community and family perception | - Community awareness | - Will improve if the study is well advertised for - Stress on the benefits to the country and to the future generations |
| Motivations | - Incentives | - Money is not advisable (wealthy participants) - Events, gatherings - Salon coupon: they will participate but they will not follow up - Appreciation certificate |

**Analysis (Transcript 2- NYUAD Staff G2)**

| **Themes** | **Categories** | **Codes** |
| --- | --- | --- |
| Perception of medical research | - Reasons for participation in medical research - Reasons for refusal of participation in medical research | - Uniqueness of this type of research which was not done previously in the UAE - Curiosity - Personal Connections, family connections - Desire to contribute in educating others - Interest in topic - Understanding of the effect of the research on society and the UAE - Benefit to participants - Being educated - Time constraint - No immediate reward - Not addressing the benefits of the study to society and the UAE - No feedback on blood tests |
| Publicity | - Increase community awareness | - Importance of involving awareness - Communicating: taking research to a different level - Advertising and encouraging UAE research - “Importance of facts and figures. E.g. in 1970 – the figures were... Then in 1990, number rose to... Underneath ‘together we can stop this increase in number’”. |
| Research design (cohort) | - Follow up - Drop outs | - Based on the culture of universities, the reason people did research was for their degree; “it would take time to instil the sense of follow up” - Emphasis on the importance of the research purpose and its implications on the understanding of diseases - Having UAE research databases - Giving updates on research results - length of study - discomfort in undergoing multiple examinations and checkups - losing interest - discomfort in sharing too much information |
| Confidentiality | - Breach of confidentiality | - Majority support of access to medical records as long as participant is not identified. |
| Genetic research |  | - People are aware of genetic research - The establishment of the Dubai Center for Genetic Research - No objections to conducting genetic research - People who may not know why their blood samples are taken will be questioning genetic research |
| Names and slogans | - Stimulating an action - Shedding light on uniqueness of study - Indicative of seriousness and commitment | - Suggestions listed are overused words; do not direct the notion that health is the target - “Do it now for the future of your children” - “Are you someone who wants to participate in preventing diseases?” - “Do you want to take part in eradicating diabetes in the UAE?” - “Our lives” - “You are making a difference” - “This is very honorable for you” |
| Participants Information Leaflet | - Adequacy and clarity of information - Level and clarity of language - Length of the process and repetition of content - Use of social media | - Usage of direct and comprehensive information in the invitation E-mail - Email title is too long - Appearance of logo images instead of listing collaborating organizations - Arabic language clarity is needed - Inadequate language on Risks - Inappropriateness of including natural risks associated with giving blood like fainting, bruising and contamination - In the second paragraph on risks, the text would “put people off” - Combining of sections - Providing a summary on a concise guidance to the study and its protocol - Repetitive content - Rearrangement of content - Easy access of Social media |
| Study process | - Explicit information of participant contribution to the society - Emphasis on research in the UAE | - Detailed and clear - Important to include how participants are contributing - Important of having research data on the UAE population |
| Expectations | - Factors associated with expected positive experiences - Factors associated with expected negative experiences | - The research is not like other research done in the UAE. - The research is one of its kind - Introducing the study in an unprecedented approach - Focus on prevention of disease - Address benefits of study on population - No immediate reward - With storing biological samples indefinitely, people may be unaware of the problems that may happen - No feedback of results |
| Community and family perception | - Diseases in the family - Awareness of the problem | - People are aware that obesity, diabetes and heart disease is a problem in the UAE. - Family members have been diagnosed with such diseases. |
| Motivations | - Incentives | - Patriotism - Honor of contributing; Certificates of Appreciation - Bias to data - May incline withdrawal |

**Analysis (Transcript 4- NYUAD Staff G4)**

| **Themes** | **Categories** | **Codes** |
| --- | --- | --- |
| Perception of medical research | - Reasons for participation in medical research - Reasons for refusal of participation in medical research | - Past experience - Family history - Knowledge of the topic - Helps research and understanding - Monetary incentives - Motivation to stay healthy - Awareness of research - “we are underrepresented in scientific research” - Conformity with the majority - Lack of awareness of the importance of research - Privacy is invaded when participant name linked to results - Lazy - Busy - Refusal to know about their personal health status |
| Publicity | - Increase community awareness - Personal connection - Legitimate messages - Enlisting potential benefits to society | - When the blood donation bus comes, talk to people about the research a month in advance so that they are ready - Talk to potential participants in a major campaign to spread awareness and knowledge of the study   - Fitness gyms   - Spas   - Universities - Handout leaflets in hospitals - “Get people who are very prominent in twitter to publicize the study” - Awareness of the diseases |
| Research design (cohort) | - Follow up | - Dissemination of results to participants would motivate follow up - The use of phone calls, Email, instant messages in follow up if effective - Extra effort needed to instil follow up procedure |
| Confidentiality | - Breach of confidentiality | - Participants are not likely to join the study if their name is linked to the results |
| Genetic research | - Concerns - Awareness - Blaming diseases on genetics - Lack of understanding of what genetic research really is - The benefit of genetic research on the etiology and prevention of diseases | - “People blame obesity [and] a lot of things on genetics without understanding what they think of genetics” - “There is not much understanding of genetic research - People had a concern with genetic research - There is awareness of genetic diseases more than before - “Better understanding of genetics would help make conditions [like Autism] less prevalent” |
| Names and slogans |  | - Clear idea - Nice and catchy word - Combines time and health - Name would have to reflect the purpose of the study - Slogan “ this is going to increase understanding of how future generations can be healthy” |
| Invitation Email and Participant Information Leaflet | - Assertiveness - Passive language - Rearrange order of subheadings - Rearrange order of contents in subheadings - Repetition of content - Statistics - Reader’s attitude | - Email title is very long - Suggested title: “Invitation: Participant information” - Better organization is needed - Long texts for reading - Present collaborating institutions in logos - Use of assertive and confident words instead of passive language - Place positive information before negative information - Negative information affects reader’s attitude - Place confidentiality statement before risks - Prevalence of diseases in the population - Section on risks (second paragraph), “would definitely turn some people away from the study” |
| Study process | - Use of biological samples - Feedback of results | - Agreement to store biological samples indefinitely for research purposes - Feedback of results would not turn people away from study |
| Expectations | - Factors associated with expected positive experiences - Factors associated with expected negative experiences | - Seeing real statistics in the future - Knowledge of the health state of the UAE and Abu Dhabi - “It is necessary to have valuable information about our health” - Updates on the study and knowledge of results - Liaise with the Ministry of Health - Promoting the research through media, newspapers in advance - Extra effort and time needed to increase both awareness and participation - Lack of awareness of research amongst older generation of participants - Participants giving consent would skip reading important information about the study - Hesitant to share information about health topics other than diabetes, heart disease and obesity - Struggle to increase participation rate due to not liaising with Ministry of Health or HAAD |
| Community and family perception | - Research culture - Family attitudes - Community awareness | - Lack of knowledge of research - Lack of understanding of causes of diseases - Educated people are more likely to understand the impact of medical studies - Conservative families may influence participation of women in the study due to the presence of men - Research is new to the Western region: people have not participated in medical research before - Community awareness that “this is going to help me and all the people in my community” - It is easier to reach Abu Dhabi city than the Easter or Western regions |
| Motivations | - Incentives - Satisfaction - Personal stories - Role models | - Honor participants - Certificate of appreciation - Personal stories and experiences - Popularity of study - Stimulating a good feeling in participation - Benefit to society stimulates satisfaction - Involving role models would increase participation   - Leaders, government leaders   - Leaders of large companies   - Prominent people in society   - Popular football players   - Supporters of the role of women in the UAE |
| Socio-economic status | - Poor - Luxury | - Social support for widows - Housing provided for the divorced - “If you are poor you will never be homeless. You will never be cold or hot” - People would stay in a farm or the desert for a vacation, there will be electricity and water - Definition of poor: people who “don’t afford luxuries that people have here” - Luxuries are: latest smartphone, going to private schools, going to private universities” |

**Analysis (Transcript 5- Blood bank G5)**

| **Themes** | **Categories** | **Codes** |
| --- | --- | --- |
| Perception of medical research | - Reasons for participation in medical research - Reasons for refusal of participation in medical research | - Confirming a theory - Prevention of disease - Improvement of:   - knowledge of disease   - quality of life based on evidence   - new discoveries - Conducting hypothesis and research questions - Length of time - Busy - Lazy - Lack of knowledge of the importance of research and the magnitude of the problem - Lack of knowledge of research plan - Cultural reasons - Social reasons |
| Publicity | - Increase community awareness - Awareness on importance of research - Awareness on diseases - Importance of Follow up - Delivering messages of video | - Educating the public on details of the study - Educating the public on complications and dangers of the diseases - Educating the public on the importance of follow up - Impact of participation to the research - Using media to publicize and promote the importance of research - Importance of the study to the society - Using TV, Radio, Social Media: Twitter, Instigram, YouTube - Attractive and interesting methods to deliver message through video:   - Comic   - Short (2 minutes) - Involving nationals with popular accounts - Establishing “friends of the study” - Gain popularity through stating big - Re-Tweet by UAE leaders - Events, invitation to participants and their families:   - Barbeque   - Poetry event   - Theatre   - Parks   - Health awareness days   - Entertainment events - General health check-ups in events |
| Research design (cohort) | - Follow up - Drop outs | - Participants willingness for follow up - Higher chance of follow up as blood donors donate more than once a year - Convinced about importance of research - Sending reminders through phone calls and messages - Participation by own will and interest - Educating participants that the effort put in participating would benefit society and future generations - “Cause too much work” - Distance to assessment center - Imposing participation - Excitement decreasing over time - Moving locations |
| Confidentiality | - Clarification on confidentiality statement | - Ensuring Privacy and confidentiality - Securing private information from researchers - Accessing the medical records is accepted   - will replace direct disclosure of information from participants   - involvement of Health Authority is trusted - Accessing the medical records is not accepted due to:   - privacy,   - shyness,   - medical records are best kept with physicians |
| Genetic research |  | - More acceptability of new generations - Genetic research would benefit society as marriages within the family are common - “Individuals would love to know if there is any risks or if there [are] any genetic diseases running in his family” - Knowledge about genetic research will be life preserving - The benefit of genetic research helps avoid future diseases - Lack of knowledge on why and how genetic diseases appear - Majority of people will agree to genetic research, minority will disagree |
| Names and slogans |  | - “Ajyaluna”: targets youth and older generation; many generations, implies tomorrow - “Hayatuna: anything that is ours because it will include everybody”, implies now - “Khutwati: a step for them they will feel involved” - صحة أفضل لمجتمعنا - أجيالنا أمانتنا - صحتنا أمانتنا - لأجل أجيال تنعم بالصحة - Emarati accent/poem |
| Participants Information Leaflet | - Time spent reading information - Adequacy and clarity of information - Level and clarity of language - Extent to which characterization of risks is clear - Instilling fear of study participation - Length of the process and repetition of content - Undue pressure to enroll - Adaptation of information to the individual - Use of video media |  |
| Study process | - bio banking | - Bio banking of biological samples is accepted by all members |
| Expectations | - Factors associated with expected positive experiences - Factors associated with expected negative experiences | - Effect of the research on participants, their families and their children in the future - Uniqueness of research - Opportunity for improvement by comparison with previous research - Opportunity for development by answering questions about health - Popularity can change people’s opinion to participate - Conservative societies “won’t disclose any personal information even for the researcher” - Bias due to involvement of leaders - No feedback of general results - No personal benefits |
| Community and family perception | - Community awareness | - Increase knowledge about the importance of the research - Promote value of participation - Creating a good health platform in the UAE - Children influence on parents |
| Motivations | - Role models - Incentives for follow up - Trust | - UAE National Role models:   - Sheikh Mohamed Bin Zayed   - Sheikh Abdulla Bin Zayed   - Educated people   - Actors   - Poets   - Writers   - Religious experts   - Comedians     - Abdul Azeez Bin Baz - Discounts ie. on hotels - Involving Ministries - Involving government authorities - Continuation of promotion and excitements |

**Analysis (Transcript 6- Blood bank G6)**

| **Themes** | **Categories** | **Codes** |
| --- | --- | --- |
| Perception of medical research | - Reasons for participation in medical research - Reasons for refusal of participation in medical research | - Feasibility study is important : effectiveness of the results on the society - To know the risk factors of a disease, diagnostic issues and new treatments - To understand the importance of research in identifying and addressing future directions - Social policies, health policies, educational policies - development of our future and the future of our societies and the future of our generations - not understanding the importance of these studies and their impact which may lead them to not participate. - They may not understand the tangible results of the study. - people are traveling, - the questionnaire is too long, - bored from the issue, - people may die - due to the organization of the study - the logistics - the timing, how the procedure fits to my personal schedule, as participation is voluntary |
| Publicity | - Increase community awareness - Awareness on importance of research - Awareness on diseases - Importance of Follow up - Delivering messages of video | - Advantages of the study before anything - Inform people that there will not be direct benefits - Benefits for children and grandchildren in future - Marketing and TV - Clarity in what the study is about |
| Research design (cohort) | - Follow up - Drop outs | - Distribution: - Near to residential areas - Ambulatory health centers (SEHA) - Private clinics - Importance of the sequence - Less waiting time at the clinics - Sending the survey before attending the clinic - Shorten the time in the clinics (less than one hour) - People must be committed to follow up - Clarity of what the study is about |
| Genetic research |  | - Genes are like imprint - Differences:   - from the actual samples (metabolism disorders different from genetic disorders)   - characteristics and ethics - Genes are special and unique to the area - Difficult to generalize it - Linked to an area or tribe or family - Possibility of a disease - Concerning of telling the family of a possible disease |
| Names and slogans | - Names - Slogans - Logos | - Ajyaluna and Hayatuna - positive - Ajyaluna: - special - people will hold it more - معاً نحو مستقبل صحي أفضل - حياة صحية أفضل لنا ولأجيالنا - Local - Logos became a trend - Visual - First impression - Cartoon: Father and son holding hands - Adding health |
| Participants Information Leaflet | - Time spent reading information - Adequacy and clarity of information - Level and clarity of language - Extent to which characterization of risks is clear - Instilling fear of study participation - Length of the process and repetition of content - Undue pressure to enroll - Adaptation of information to the individual - Use of video media | The e-mail:   - The beginning is nice : give you a trust and honesty (دعوة للمشاركة...) - Rephrasing **لابد** من قراءة المحتوى - Adding brackets:   بالتعاون مع: شركة أبو ظبي للخدمات  الصحية (صحة): ( مدينة الشيخ خليفة الطبية، وبنك الدم)، مستشفى زايد العسكري، جامعة الإمارات العربية المتحدة، وجامعة زايد.   - Marketing : website or logo - No logos: Some people like to read more |
| Study process | - bio banking | - “It is okay to review medical records however, others may not agree. Some health conditions people are willing to share and there are health conditions that people are not willing to share.” - “The consent and agreement of the participant is very important” - “I see that designating a specific time in which my medical records are reviewed is very important. If I give agreement now it is different than if I give agreement after 10 years.” |
| Expectations | - Factors associated with expected positive experiences - Factors associated with expected negative experiences | - “Seeing what time is most suitable for the participants. This kind of procedure makes me feel that yes, I would love to be part of the study process.” - The subject is positive heart and soul - Has many positives - Important study - Importance of the budget - Logistics - Communication to the participants - Participants arrivals to the clinics - Participant’s priorities and time - The research needs work from the research team : storing and transporting samples |
| Motivations | - Positives and Negatives | - Money Kills the subject and the belongings to the study - Sensitivity toward money - Acceptance of the money for time spending - Morale rather than materialism |
